# Preface: Stem cells in non-human primates

**DOI:** 10.5194/pb-4-241-2017

**Published:** 2017-12-19

**Authors:** Rüdiger Behr

**Affiliations:** German Primate Center, 37077 Göttingen, Germany

Among the most relevant areas of biomedical research are novel insights into human diseases associated with the degeneration of specific tissues and organs and how to treat these. Stem cells may prove to be of extraordinary value for the understanding and treatment of pathological processes. For the investigation of pathological processes in humans, patient-specific-induced pluripotent stem cells are a very useful and promising tool, often allowing the *modeling* of the disease in a cell culture dish. In fact, numerous exciting insights into pathomechanisms have been obtained in recent years by in vitro studies of human cells. However, translational approaches to *curing* diseases in the context of living organisms are very complex and can – for ethical reasons – not be performed in humans without previous careful and serious assessment of the risks and potential benefits of such therapies. Among those diseases that may be treatable by stem-cell-based therapies are Parkinson's disease, macular degeneration, myocardial degeneration, and infertility. Many other diseases may also be treated in the future using different types of stem cells. However, particularly pluripotent stem cells and germ cells have the potential to form specific types of tumors called teratomas. Tumor formation, however, would be an unacceptable side effect. Moreover, stem cells may be attacked by the recipient's immune system. Hence, it is important to identify minimal but efficient immunosuppression protocols for stem-cell-based therapies. Finally, the functionality of cells and tissues derived from transplanted stem cells (i.e., the efficacy of the treatment) needs to be evaluated in the context of closely resembling the clinical situation without putting humans at risk of experimental therapies. Here, non-human primates (NHPs) come into play. They are very valuable animal models in translational biomedical research due to their close physiological, anatomical and genetic resemblance to humans. Consequently, NHP stem cells need to be generated, investigated and subsequently tested in regenerative therapies in vivo to promote translation into clinical application.

This special issue of *Primate Biology* on NHP stem cells covers different aspects of NHP stem cells. Wianny and Vezoli provide an excellent overview of the current state of research using a NHP model of Parkinson's disease and the stem-cell-based approaches to curing this medically and socio-economically highly relevant neurodegenerative disease. Sharma and colleagues discuss the characteristics of male germ-line stem cells in NHPs and show the relevance of primate research by highlighting the discrepancies between primate and mouse germ-line stem cells. This is particularly true from the perspective of male germ-line stem cells, which may be used in the future for the treatment of male infertility. Hemmi and colleagues investigated the age effect of cell donors on the efficiency of the generation of induced pluripotent stem cells and their neuronal differentiation potential. This is of major importance since the primary risk factor of most degenerative diseases in humans, including Parkinson's disease, is age. Hence, efficient iPS cell generation from older persons is important. Finally, Rodriguez-Polo and colleagues describe for the first time the expression of a tyrosine kinase, c-CBL, in pluripotent stem cells. They used the marmoset monkey as a NHP model. Since mutated c-CBL plays a role in myeloid malignancies, it is reasonable to assume that this tyrosine kinase also plays an important role in pluripotent stem cells.

We hope that this special issue of *Primate Biology* further promotes NHP stem cell research and that it highlights the translational value of non-human primates in this exciting and highly topical field of research.

